# Nivolumab-induced diabetes mellitus—a case report with literature review of the treatment options

**DOI:** 10.3389/fimmu.2023.1248919

**Published:** 2023-10-26

**Authors:** Eveline Daetwyler, Alfred Zippelius, Simona Danioth, Marc Y. Donath, Lara Gut

**Affiliations:** ^1^ Department of Medical Oncology, University Hospital Basel, Basel, Switzerland; ^2^ Department of Biomedicine, University of Basel, Basel, Switzerland; ^3^ Clinic for Endocrinology, Diabetes & Metabolism, Luzern Cantonal Hospital, Luzern, Switzerland; ^4^ Clinic for Endocrinology, Diabetes & Metabolism, University Hospital Basel, Basel, Switzerland; ^5^ Clinic for Endocrinology & Diabetes, Medical University Clinic Baselland, Liestal, Switzerland

**Keywords:** immune checkpoint inhibitor (ICI), immune-related adverse event (irAE), diabetes mellitus, TNF blockade, case report

## Abstract

**Background:**

Immune checkpoint inhibitor (ICI) treatment has become important for treating various cancer types, including metastatic renal cell carcinoma. However, ICI treatment can lead to endocrine immune-related adverse events (irAEs) by overstimulating the patient’s immune system. Here, we report a rare case of a new onset of diabetes mellitus (DM), caused by nivolumab, and we discuss the feasible treatment options with a focus on TNF antagonism.

**Case presentation:**

A 50-year-old man was diagnosed with metastatic renal cell carcinoma. Due to systemic progression, a combined immunotherapy with ipilimumab and nivolumab was initiated, according to the current study protocol (SAKK 07/17). The administration of ipilimumab was stopped after 10 months, due to partial response as seen in the computer tomography (CT), and nivolumab was continued as monotherapy. Fourteen months after the start of the treatment, the patient was admitted to the emergency department with lethargy, vomiting, blurred vision, polydipsia, and polyuria. The diagnosis of DM with diabetic ketoacidosis was established, although autoantibodies to β-cells were not detectable. Intravenous fluids and insulin infusion treatment were immediately initiated with switching to a subcutaneous administration after 1 day. In addition, the patient received an infusion of the TNF inhibitor infliximab 4 days and 2 weeks after the initial diagnosis of DM. However, the C-peptide values remained low, indicating a sustained insulin deficiency, and the patient remained on basal bolus insulin treatment. Two months later, nivolumab treatment was restarted without destabilization of the diabetic situation.

**Conclusions:**

In contrast to the treatment of other irAEs, the administration of corticosteroids is not recommended in ICI-induced DM. The options for further treatment are mainly based on the low numbers of case series and case reports. In our case, the administration of infliximab—in an attempt to salvage the function of β-cells—was not successful, and this is in contrast to some previous reports. This apparent discrepancy may be explained by the absence of insulin resistance in our case. There is so far no evidence for immunosuppressive treatment in this situation. Prompt recognition and immediate start of insulin treatment are most important in its management.

## Introduction

Immune checkpoint inhibitor (ICI) treatment has become an important therapeutic option in the first-line treatment of metastatic renal cell carcinoma (1–4). While being effective, ICI treatment is associated with a broad spectrum of autoimmune complications, known as immune-related adverse events (irAEs) (5–7). Thus, ICI-induced diabetes mellitus (DM) is a rare side effect but develops with a rapid loss of insulin production (5–7). Accordingly, it often presents clinically with an acute onset with severe and persistent insulin deficiency (8, 9). In this report, we present a case of nivolumab-induced DM, presenting with diabetic ketoacidosis (DKA).

## Case presentation

A 50-year-old man was diagnosed with metastatic renal cell carcinoma in 2016 which was initially treated by local pulmonary surgery (2016, 2017) and 3 years later with local excision of a metastatic lesion of the right thigh (2019). Four years after the initial diagnosis, the disease progressed at multiple sites (2020). Therefore, the patient was enrolled in the study SAKK 07/17 (10), including a combined immunotherapy with ipilimumab, a CTLA-4 inhibitor, and nivolumab, a PD-1 inhibitor. Ipilimumab was administered every 6 weeks with a dosage of 1 mg/kg body weight intravenously, beginning 2 weeks after the start of nivolumab. Nivolumab was administered every 4 weeks with a dosage of 480 mg intravenously. The administration of ipilimumab could be stopped 10 months after the beginning of the treatment according to the study protocol, as a result of partial response in the CT, and nivolumab was continued. The treatment was well tolerated, and no abnormalities of blood glucose were noted so far in a normal-weight patient (BMI 24.9 kg/m^2^).

However, 17 days after the last cycle of nivolumab and 14 months after the start of the whole treatment, the patient presented to the emergency department with lethargy, vomiting, blurred vision, polydipsia, and polyuria.

### Diagnostic assessment

Laboratory testing revealed DKA with a venous blood gas pH of 7.058 and serum glucose of 29.0 mmol/L (**Table 1**). The urine analysis was positive for glucose, ketones, and proteins. The level of HbA1c was elevated to a value of 8.7% and C-peptide was low (<50 pmol/L), indicating an insulinopenic DM. The serum titers of anti-glutamic acid decarboxylase (anti-GAD) and anti-tyrosine phosphatase (anti-IA2) antibodies were not detectable. The values of amylase and lipase were remarkedly elevated without clinical signs of pancreatitis. The patient had no symptoms of exocrine pancreas insufficiency; therefore, the pancreatic elastase in the feces was not measured. Interleukin 6 was elevated in the context of a normal CRP. There were no signs of hypophysitis or other irAEs. In magnetic resonance imaging of the pancreas, there were no signs of pancreatitis or tumor progression in the pancreas. The findings were interpreted in the context of a new ICI-induced DM with the main symptom of a DKA.

**Table 1:** Laboratory results on day 1 of admission (in blue = values below the threshold value, in red = values above the threshold value, in black = values in the normal range).

Analysis	Value	Reference range
Venous blood gas Ph	7.058	7.38–7.42
Serum glucose (mmol/L)	29.0	4.3–6.4
Sodium (mmol/L)	131	135–145
Potassium (mmol/L)	5.3	3.6–4.8
Bicarbonate (mmol/L)	8.3	21–26
Anion gap (mmol/L)	29	8–16
Pancreatic amylase (U/L)	318	13–53
Lipase (U/L)	402	21–67
CRP (mg/L)	<5	<5
Interleukin 6 (ng/L)	15.2	<7.0
HbA1c (%)	8.7	4.8–5.9
Anti-GAD IgG (IU/ml)	<10	<10
Anti-IA-2 IgG (U/ml)	<15	<15
C-peptide (pmol/L)	<50	370–1,470

### Therapeutic intervention

**Figure 1:**
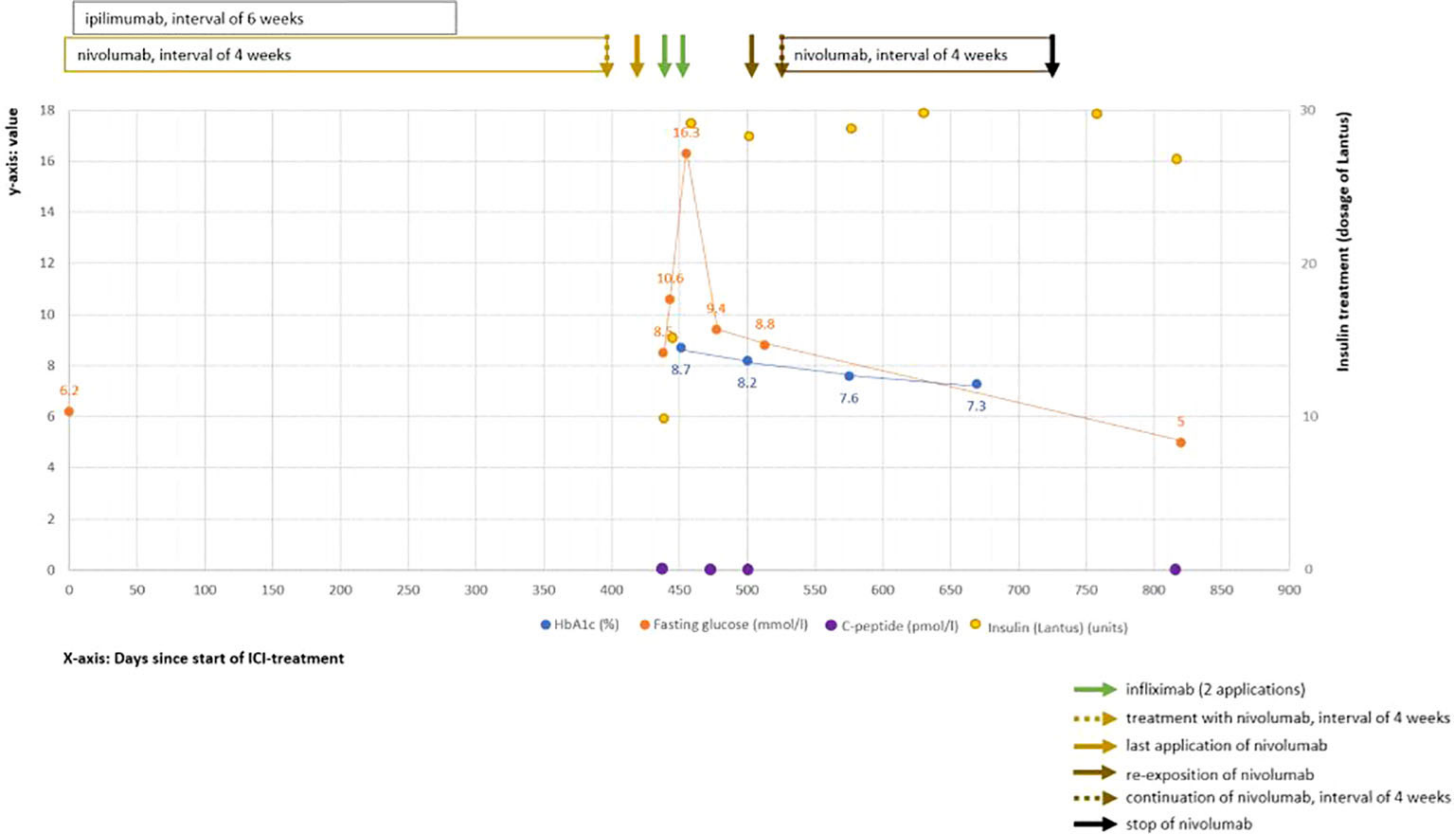
Clinical course of ICI-induced diabetes mellitus. Shown are values over the time course after starting ICI treatment (time point 0). The *Y*-axis (left side) shows the laboratory values of HbA1c (in blue color, %), fasting glucose (in orange color, mmol/L), and C-peptide (in violet color, pmol/L). On the right *Y*-axis, the insulin dosages (in yellow color) are displayed. The *X*-axis shows the time interval since the start of the ICI treatment in days.

The patient was referred to the intensive care unit (ICU), and intravenous fluids and continuous insulin infusion treatment were immediately initiated. After 1 day, ketoacidosis was resolved and glucose levels were improved; thus, insulin therapy was switched to a subcutaneous administration of insulin glargine (Lantus) and insulin lispro (Humalog). In an attempt to preserve the remaining β-cell function, infliximab (5 mg/kg body weight) was additionally administered on the fourth day of hospitalization with a repetition in the same dosage after 2 weeks. These applications were well tolerated by the patient. The C-peptide values remained low after the second infliximab infusion. Accordingly, the treatment with infliximab was not continued. The patient could be discharged with satisfactory glucose levels with insulin glargine (28 units per day) and insulin lispro (sliding scale). Over the following year, the patient remained on multiple daily insulin injections with unchanged insulin requirement. He received a flash glucose monitoring system and instructions from the local diabetes department resulting in an improvement of HbA1c and fasting glucose levels (**Figure 1**). Nevertheless, the patient still showed significant glucose fluctuations similar to type 1 DM. Additionally, the C-peptide values remained low (**Figure 1**).

### Follow-up and outcome

Two months after the diagnosis of an ICI-induced DM, nivolumab was restarted without destabilizing the diabetic situation. In the recent CT scan, the patient showed persistent partial response. The treatment with nivolumab could be stopped after 2 years of treatment according to the study protocol within a still ongoing partial response in the CT.

## Discussion

DM is a rare irAE after ICI treatment. It occurs in approximately 0.1%–1.0% of patients treated with ICI (8, 9). In a large case series, most cases occurred after the application of monotherapy with a PD-1 inhibitor (76%), followed by a combination therapy of CTLA-4 with either PD-1 or PD-L1 (17%) (11). ICI-induced DM is diagnosed at a median of 7–25 weeks after initiation of ICI treatment. Risk factors for an early manifestation of this irAE are described in patients with DKA (9, 12), documentation of positive islet cell antibodies (9, 12), and with a combined PD-1 and CTLA-4 inhibitor treatment.

To date, only limited data are available regarding pathophysiology and disease definition. In general, ICI-induced DM is defined as severe and persistent insulin deficiency presenting as i) DKA or with decreased to absent C-peptide and ii) persistent insulin dependence for at least weeks to months after acute illness (8, 13). However, its differentiation from an initial presentation of type 1 DM can be particularly difficult.

ICI-induced DM presents acutely in 50%–75% of cases in the setting of DKA (8, 9, 13, 14) as also shown in our case report. However, a decreased incidence of DKA over the last years could be observed mainly due to increased awareness leading to an early detection and initiation of treatment (14). As the HbA1c is often elevated to 7.6%–9.7% at diagnosis, subacute severe hyperglycemia must be assumed (9, 13, 14). However, due to the commonly rapid onset of hyperglycemia in ICI-induced DM, HbA1c is not a reliable screening parameter in this patient population (15). In the case series of Stamatouli et al. (9), in 85% of patients, it was shown that acute progressive hyperglycemia is due to rapid β-cell loss, evident from low or even absent C-peptide values at diagnosis. This hypothesis is supported by the case series of Tsang et al. (14), in which 6 out of 10 patients who developed ICI-induced DM had detectable C-peptide levels shortly before diagnosis, with a marked decrease or complete absence of detectability shortly after diagnosis. These results contrast with the observations in the Type 1 Diabetes TrialNet, where 93% of patients with type 1 DM still showed detectable C-peptide levels 2 years after initial diagnosis (16).

Additional markers in DM are DM-specific antibodies. A relevant proportion (40%–70%) of patients with ICI-induced DM have at least one positive DM-specific antibody (most anti-GAD) (9, 13, 17). However, their clinical and diagnostic relevance has not yet been fully clarified. In comparison, the autoantibody positivity in type 1 DM is 70% for anti-GAD (18), 58% for anti-IA2 (12), and 60%–80% for anti-zinc transporters, respectively. As in spontaneous type 1 DM, ICI-induced DM may be based on a genetic predisposition, with HLA genes being of particular importance as they are significantly associated with the occurrence of many autoimmune diseases. In several case series (8, 9), a predominance of DR4 and DR3 alleles could be shown in patients with ICI-induced DM, known also as susceptible alleles for the development of type 1 DM. On the other hand, the presence of protective HLA genes did not prevent the occurrence of ICI-induced DM (14).

Interestingly, elevated pancreatic enzymes can be additionally detected in 32%–57% at diagnosis, with imaging demonstrating pancreatic lesions in some cases (9, 11, 13). This suggests that the diagnosis may be preceded by exocrine pancreas inflammation. Case series support this hypothesis and show the presence of acute pancreatitis in 20% of cases at diagnosis.

Even though ICI-induced DM seems to be pathophysiological based on an insulin deficiency and thus has similarities with type 1 DM, the median age of disease manifestation (62 to 68 years) is significantly higher than in patients with type 1 DM or even LADA (latent autoimmune diabetes in adult) (9, 13, 14). This can be seen in the context of ICI exposure at older ages. As the median BMI (26 to 32.2 kg/m^2^) is higher than would be expected in type 1 DM (9, 13), the differentiation from exacerbation of underlying type 2 DM may also be difficult unless there was a documented, well-controlled type 2 DM without insulin administration before the initiation of ICI treatment.

Due to the frequently acute manifestation of the disease with associated high morbidity for this already at-risk population and the rising incidence with increasingly widespread use of ICIs, thus, early detection of ICI-induced DM is of particular importance. Therefore, the ASCO Guidelines (7) recommend regular monitoring of plasma glucose before and at the beginning of each therapy cycle as well as during follow-up for at least 6 months. If an ICI-induced DM is suspected, the international guidelines (ASCO, ESMO, SITC) recommend a diagnostic workup including the examination of plasma glucose, HbA1c, diabetes-specific antibodies, C-peptide, anions gap in the metabolic panel, and urinary ketones (5–7, 11). After diagnostics, therapeutic measures should be initiated promptly and not be delayed pending the results.

For the management of ICI-induced DM, the current international guidelines (ASCO, ESMO, SITC, ESE) recommend treatment with insulin. If DKA is present, hydration, the use of insulin perfusion, correction of the electrolyte abnormalities, and ICU monitoring are mandatory (5–7, 15, 19).

In almost all reported cases, insulin dependency in ICI-induced DM was permanent. To our knowledge, only in three cases with ICI-induced DM could insulin treatment be stopped in the further course of the disease. Trinh et al. reported a case of ICI-induced DM with positive autoantibodies against islet cells, impaired insulin secretion, and insulin resistance where insulin treatment could be stopped after infliximab and intra-articular corticosteroid injections administered due to an oligoarthritis (20). In the second case, β-cell function could be regained after stopping pembrolizumab therapy, resulting in an improvement of glycemic control and detectable C-peptide values. Unfortunately, no baseline C-peptide was measured at the time of diagnosis, and therefore, it is not clear whether this patient was insulinopenic and fulfilled the diagnostic criteria of ICI-induced DM (21). The third case is a patient with BMI 26 kg/m2 (2), pre-existing hypertension, and dyslipidemia, with a detectable C-peptide at the time of diagnosis (1.0 nmol/L, normal value >0.37), a high HbA1c of 11.4%, and without DKA at the time of presentation (22). In all three cases, an insulin resistance was identified or may be suspected which might explain the different course of these cases compared with our case. Additionally, the lack of islet autoantibodies in our case may point to a difference in etiology.

In addition, there have been various attempts to treat ICI-induced DM with glucocorticoids since this treatment is well established for other irAEs. None of these attempts resulted in the resolution of the DM or reduction of insulin dosage (9, 13, 19, 23–27). Therefore, ESMO (5), ASCO (7), and ESE Guidelines (15) do not recommend the use of glucocorticoids in ICI-induced DM (**Table 2**).

**Table 2:** Evaluated systemic therapeutic options for ICI-induced DM in addition to insulin treatment.

Case series and case reports			
Treatment	Glucocorticoids	Glucocorticoids + GLP-1 agonist	Infliximab + intra-articular corticosteroid infiltration
**Author (number of patients)^reference^**	1. Aleksova et al. (*n* = 1) (23) 2. Stamatouli et al. (*n* = 4) (9) 3. Kapke et al. (*n* = 1) (24) 4. Chae et al. (*n* = 1) (25) 5. Porntharukchareon et al. (*n* = 1) (26)	Fukui et al. (*n* = 1) (27)	Trinh et al. (*n* = 1) (20)
**Dosage**	1. Prednisone 2 mg/kg body weight 2. Prednisone 50 mg daily (*n* = 1) 10 mg daily (*n* = 3) 3. Prednisone 60 mg daily 4. Prednisone 10 mg daily 5. Prednisone 7.5 mg daily	Prednisone 1 g daily Exenatide 10 μg daily	No information
**Insulin treatment**	Persistent insulin treatment	Persistent insulin treatment	Insulin stop
**Effect on β-cell function**	All studies: no effect	No effect	Reversal of β-cell dysfunction Remark: partial insulin resistance
International guidelines			
**ASCO Guidelines 2021 (** 7)	(X) Not indicated	(Not applicable) No statement	(X) Not indicated
**ESMO Guidelines 2022 (** 5)	(X) Not recommended	(Not applicable) No statement	(Not applicable) No statement
**SITC Guidelines 2021 (** 6)	(Not applicable) No statement	(Not applicable) No statement	(Not applicable) No statement
**ESE Guidelines 2022 (** 15)	(X) Not recommended	(Not applicable) No statement	(Not applicable) No statement

Other immunosuppressive agents such as infliximab have been considered for the treatment of ICI-induced DM. This is based on the fact that TNF-α plays an important role in insulin resistance in rodents and that TNF-α blockers had a beneficial effect in limited cases of type 1 DM (28–31). Motivated by this and the case report by Trinh et al. (20), we opted for an early infliximab therapy in our case which unfortunately did not result in the preservation of the remaining β-cells. A possible explanation for the treatment failure of infliximab may be the absence of insulin resistance in our case, which was present in the case of Trinh et al. (20)

Additionally, other immunosuppressive treatments have been tested in ICI-induced DM. Hereby, some immunosuppressive agents (e.g., abatacept, CTLA-4-Ig) that are tested in type 1 DM are not suitable for ICI-induced DM as they interfere with the T-cell reaction necessary for the antitumor reaction. Other agents, such as tocilizumab (anti-IL-6) or rituximab (anti-CD20), have been described as prolonging C-peptide production without interference in antitumor immunity and therefore might influence β-cell dysfunction in early ICI-induced DM (8, 32, 33). To our knowledge, there are no data regarding these immunosuppressants in ICI-induced DM (**Table 2**).

Moreover, it needs to be considered that—by the time of a manifest hyperglycemia in type 1 DM—already 40%–95% of the pancreatic β-cells are irreversibly lost with possibly an even greater loss in ICI-induced DM due to its rapid occurrence (34–36). Therefore, it is questionable whether any immunosuppressive treatment may preserve β-cell function, even if administered early. Due to this fact, the current international guidelines do not indicate the use of any immunosuppressive treatment (7).

Regarding the ICI treatment itself, international guidelines recommend pausing it until glucose levels are controlled or at least until DKA is resolved (5–7). After stabilization of DM, current literature advocates the resumption of therapy, particularly in patients with clinical response (11, 19).

Ultimately, the treatment of ICI-induced DM consists of prolonged insulin treatment and patient education about DM management (7, 15). Assisting measures such as flash glucose monitoring may contribute to a better glucose control and HbA1c (37) as was also demonstrated by our case.

In a few studies, oral antidiabetic agents were added to the insulin regime with an improvement of glucose control despite the insulinopenic character of the DM (38, 39). Administration of a GLP-1 agonist (glucagon-like peptide-1 receptor agonist) had no influence on the endogenous insulin secretion or the insulin dosage in one case report (27) (**Table 2**).

## Conclusion

We report a case with the new onset of DM due to PD-1 blockade. The administration of infliximab did not lead to an improvement of β-cell function, which was shown in persistent low C-peptide values and the necessity of insulin injections. There is little evidence for the administration of an immunosuppressive treatment in this situation. Therefore, the mainstay of treatment remains the administration of insulin. We emphasize the need for prompt recognition, the involvement of endocrinologists, and the necessity of urgent treatment as a fatal outcome could be possible.

## Data availability statement

The original contributions presented in the study are included in the article/supplementary material. Further inquiries can be directed to the corresponding author.

## Ethics statement

Ethical approval was not required for the studies involving humans because it is not necessary (case report, retrospective). The studies were conducted in accordance with the local legislation and institutional requirements. The participants provided their written informed consent to participate in this study. Written informed consent was obtained from the individual(s) for the publication of any potentially identifiable images or data included in this article. Written informed consent was obtained from the participant/patient(s) for the publication of this case report.

## Author contributions

ED, AZ, MD, and LG participated in the care of the patient. ED, SD, and LG drafted the manuscript. AZ and MD have revised the manuscript. All authors contributed to the article and approved the submitted version.
